# RSV-infected children with mixed infections: clinical features and early predictive indicators of codetection with *Streptococcus pneumoniae* and *Haemophilus influenzae*

**DOI:** 10.3389/fped.2026.1799724

**Published:** 2026-05-04

**Authors:** Jingwen Ni, Junyu Dong, Lele Li, Mengxin Zhao, Zhihui Du, Jie Li, Kenan Fang, Kai-Sheng Hsieh

**Affiliations:** 1 Pediatric Intensive Care Unit, Luoyang Maternal and Child Health Hospital, Luoyang, China; 2 Department of Pediatric Cardiology, China Medical University Children’s Hospital, China Medical University, Taichung City, Taiwan; 3 Department of Medicine, College of Medicine, China Medical University, Taichung City, Taiwan

**Keywords:** clinical feature, *H. influenzae*, predictive indicators, RSV, *S. pneumoniae*

## Abstract

**Background:**

Since the COVID-19 pandemic, the incidence of respiratory syncytial virus (RSV) infections has significantly increased, and bacterial codetection further exacerbates the disease burden. This study aims to compare the clinical characteristics, laboratory results, and prognostic differences in children with RSV infection who are codetected with *Streptococcus pneumoniae* and *Haemophilus influenzae*, and to identify early predictive markers for such codetections.

**Methods:**

In this single-center retrospective study, we collected data from 1,601 children hospitalized with RSV infection at Luoyang Maternal and Child Health Hospital, Henan Province, between January 2023 and March 2025. Children were divided into three groups: non-bacterial codetection, *S. pneumoniae* codetection, and *H. influenzae* codetection. We compared demographic characteristics, clinical features, laboratory findings, and prognosis. Logistic regression identified risk factors for codetection of *S. pneumoniae* and *H. influenzae*.

**Results:**

A history of wheezing increased the likelihood of codetection with both bacteria. Children with *S. pneumoniae* codetection were more likely to present with fever, whereas those with *H. influenzae* codetection were more prone to wheezing and respiratory distress. The presence of extrapulmonary manifestations was a significant common factor for both codetections. Regarding laboratory markers, children with codetection of *S. pneumoniae* showed significantly elevated levels of WBC, NLR, CRP, PCT, and IL-6. For those codetected with H. influenzae, WBC, NLR, CRP, PCT, IL-6, PLT, and D-dimer levels were all significantly increased. Children with either bacterial codetection required significantly more respiratory support, had higher PICU admission rates, and experienced longer hospital stays. A history of wheezing and elevated IL-6 levels were associated with a higher likelihood of *S. pneumoniae* codetection, while younger age and higher levels of WBC, CRP, and IL-6 were predictive of *H. influenzae* codetection.

**Conclusions:**

Compared with children with RSV infection alone, those with codetection of *S. pneumoniae* or *H. influenzae* exhibit significantly elevated inflammatory markers, especially IL-6. These children are more likely to require PICU admission and respiratory support, and to experience longer hospital stays.

## 1 Background

Respiratory syncytial virus (RSV) is the leading cause of acute lower respiratory tract infections (ALRI) in infants and young children, and is one of the primary contributors to morbidity and mortality in this age group globally. Among children under five years of age, RSV accounts for approximately 3.2 million hospitalisations and 59,600 in-hospital deaths annually worldwide (1, 2). RSV infection imposes a substantial burden on healthcare systems, as well as on society and the economy. Existing studies indicate that the incidence of RSV infections has significantly increased since the COVID-19 pandemic, particularly in low and middle income countries (3). Therefore, enhancing RSV surveillance and improving early recognition of severe infections are crucial.

In recent years, two novel products for the prevention of severe RSV-associated disease have become available for infants: passive immunisation with the monoclonal antibody nirsevimab and maternal vaccination during pregnancy (4). These interventions have already begun to modify the seasonal and age-specific epidemiology of RSV in countries where they have been implemented (5). At the same time, nirsevimab has profoundly reduced RSV-associated hospitalisations, bronchiolitis severity, and PICU admission rates in immunised populations (6). However, the impact of RSV co-infection with bacterial pathogens—particularly *Haemophilus influenzae* and *Streptococcus pneumoniae*—in the context of these preventive measures remains underexplored.

Several previous studies have investigated various haematological parameters, such as the neutrophil-to-lymphocyte ratio and C-reactive protein (CRP) (7, 8), which have been evaluated as predictors of disease progression, intensive care unit admission, or prolonged hospital stay. However, these studies did not systematically assess bacterial codetection status. Importantly, codetection of *Streptococcus pneumoniae* and *Haemophilus influenzae* in children with RSV infection is associated with significantly higher hospitalisation rates, clinical severity scores, respiratory support requirements, and length of hospital stay (9). In a study of children hospitalized with bronchiolitis, those with *H. influenzae* detected in the nasopharynx had longer hospital stays and higher rates of PICU admission (10–12). Furthermore, *S. pneumoniae* and *H. influenzae* are among the most common bacterial pathogens found in RSV infections (13). As RSV infection rates rise, this study focuses on children with RSV infection who are codetected with *S. pneumoniae* and *H. influenzae*.

The aims of this study were: (1) to compare the clinical features and outcomes of children with RSV infection without bacterial codetection to those with codetection of *S. pneumoniae* and *H. influenzae*; (2) to assess the relationship between codetection with these two bacteria and worsening disease severity; and (3) to identify early predictive indicators for codetection with *S. pneumoniae* and *H. influenzae*.

## 2 Methods

### 2.1 Study design and population

This single-center retrospective study included children hospitalized in the pediatric ward of Luoyang Maternal and Child Health Hospital, Henan Province, China, between January 1, 2023, and March 31, 2025, with ALRI caused by RSV.

#### 2.1.1 Inclusion criteria

1. Age criterion: 29 days to 18 years. To fully characterise age-specific RSV burden across the paediatric spectrum and to determine whether severe disease occurs in older children, we enrolled patients aged 29 days to 18 years.
2. Diagnosis of ALRI based on established criteria (14, 15), with no use of antibiotics or probiotics in the 30 days prior to admission (16, 17).
3. Confirmation of RSV infection through antigen testing, nucleic acid testing, or metagenomic sequencing (18).

#### 2.1.2 Exclusion criteria

1. Incomplete clinical or laboratory data.
2. RSV infection acquired during hospitalization.

#### 2.1.3 Sampling method

1. Sample Collection Timing: All samples were collected as early as possible during the acute phase of hospitalization, specifically within 24 h of admission (or within 24 h of intubation for mechanically ventilated children).
2. Standard Operating Procedure:
  2.1. For children who were not intubated or mechanically ventilated, nasopharyngeal samples were collected using a sterile synthetic fiber swab.
  2.2. For children who were intubated and mechanically ventilated, endotracheal secretions were aspirated using a suction device.

#### 2.1.4 Diagnostic criteria

Extrapulmonary manifestations included neurological complications (seizures, RSV encephalopathy, RSV encephalitis, etc.) (19); cardiovascular events (acute heart failure, arrhythmias, stress cardiomyopathy, viral myocarditis, cardiogenic shock, and heart failure requiring inotropic support) (20–23); hyponatraemia (<130 mmol/L) (24); and secondary thrombocytosis (≥500 ×  10⁹/L) (25).

ALRI: Including Pneumonia, Bronchiolitis, and Acute Wheezing/Bronchitis Episodes.

### 2.2 Testing method

#### 2.2.1 Antigen detection: colloidal gold immunochromatography

The nasopharyngeal swab sample is immersed in an extraction tube for stirring and external pressure to ensure thorough soaking of the swab. After mixing, the sample is applied to the test plate, and results are read after 15 min.

#### 2.2.2 Nucleic acid testing: conventional genomic sequence RT-PCR

The collected sample is placed in a viral transport medium (VTM) and stored at 2–8°C. Within 24 h, the sample is transferred to the laboratory. Nucleic acids are extracted using the SSNP-9600A automated nucleic acid extractor (Shuoshi Biotech, Jiangsu), with an extraction time of approximately 20 min. The extracted nucleic acids are then used for downstream PCR analysis. The RSV nucleic acid detection kit (Da'an Gene) is used with real-time fluorescence PCR technology. Specific primers and fluorescent probes are designed to target the highly conserved regions of the RSV genome. One-step reverse transcription polymerase chain reaction (RT-PCR) is used to amplify the RSV RNA, and fluorescence signals are detected using a real-time PCR machine, with amplification curves plotted. The amplification time is approximately 90 min.

This method allows for simultaneous detection of RSV and other common respiratory pathogens, including *S.pneumoniae*, *H.influenzae*, *Staphylococcus aureus*, *Klebsiella pneumoniae*, *Legionella pneumophila*, and *Pseudomonas aeruginosa*.

#### 2.2.3 Metagenomic Next-Generation Sequencing (mNGS)

Pathogen nucleic acids (such as DNA and RNA) are extracted from the sample, followed by steps such as reverse transcription, enzyme digestion, and modification to construct a sequencing-ready library. The library is then sequenced using a high-throughput sequencing platform, generating a large volume of short sequence data. Bioinformatics analysis is performed to control the quality of the sequencing results, assemble sequences, identify species, and conduct functional analysis.

#### 2.2.4 Bacterial Strain Identification and Antibiotic Susceptibility Testing

Under sterile conditions, healthcare professionals collect sputum samples via nasopharyngeal or oropharyngeal aspiration. The samples are inoculated onto Columbia blood agar plates and cultured at 37°C for 24 h to promote bacterial growth. Bacterial identification is performed using the Merieux automated antibiotic susceptibility system, and antimicrobial susceptibility testing is conducted according to the Clinical and Laboratory Standards Institute (CLSI) guidelines.

### 2.3 Data collection

To ensure the accuracy of the data, this study was conducted by a team of seven clinical physicians. One physician was responsible for retrieving the list of children with positive RSV test results from the laboratory database during the study period. Two physicians reviewed and entered the clinical data of all eligible children into the electronic medical records system. Two physicians reviewed and entered laboratory test results, while the remaining two physicians were responsible for entering and verifying all the collected data. Each patient was assigned a unique research identification number to ensure data privacy, and no personally identifiable information was collected.

The primary outcomes of this study include comparisons of PICU admission rates, length of hospital stay, respiratory support requirements, and final outcomes between children with RSV infection who are codetected with *S. pneumoniae* or *H. influenzae* and those without bacterial codetection.

The secondary outcomes include the demographic information, clinical indicators (such as fever, cough, wheezing, respiratory distress, and extrapulmonary manifestations), and laboratory test results (such as WBC, NLR, HGB, CRP, PCT, SF, IL-6, PLT, ALT, AST, CK, CK-MB, LDH, BUN, Cr, UA, Na, K, Ca, PT, APTT, AT-III) for both groups.

### 2.4 Ethical statement and informed consent

This study was approved by the Ethics Committee of Luoyang Women and Children's Health Hospital (Approval No: KY2022021401.0) and registered on the Chinese Clinical Trial Registry (http://www.chictr.org.cn/index.aspx) with registration number ChiCTR2200057182. The date of Registration: 2022-10-30. Given the retrospective nature of the study and the use of anonymized patient data, informed consent was waived in accordance with institutional and national ethical guidelines.

### 2.5 Statistical analysis

The statistical analysis for this study employed a comprehensive approach to evaluate the data. Descriptive statistics were utilized to summarize the demographic and clinical characteristics of the patients, including measures of central tendency (mean or median) and dispersion (standard deviation or interquartile range) for continuous variables, and frequencies (counts) and percentages for categorical variables. The normality of data distribution was assessed using the Shapiro–Wilk test, the normally distributed continuous variables are expressed as mean ± standard deviation (SD) and compared using an independent samples *T*-test, and the non-normally distributed continuous variables are presented as quartiles with non-parametric tests, such as the Mann–Whitney *U*-test (*Z*-test statistic), were applied to compare continuous variables between different groups. For categorical variables, the chi-square test (*χ*²) was used to examine the associations between groups, with the results presented as chi-square statistics and corresponding *p*-values. To identify significant predictors of the outcomes, univariate regression analysis was first conducted, followed by multivariate regression analysis to adjust for potential confounders. The results of the regression analyses were reported as regression coefficients (*β*), standard errors (S.E.), Z-values, *p*-values, and odds ratios (OR) with 95% confidence intervals (CI). A *p*-value of less than 0.05 was considered statistically significant throughout the analysis. The median imputation (for continuous variables) and mode imputation (for categorical variables) were adopted to handle missing data.

## 3 Results

**Figure 1:**
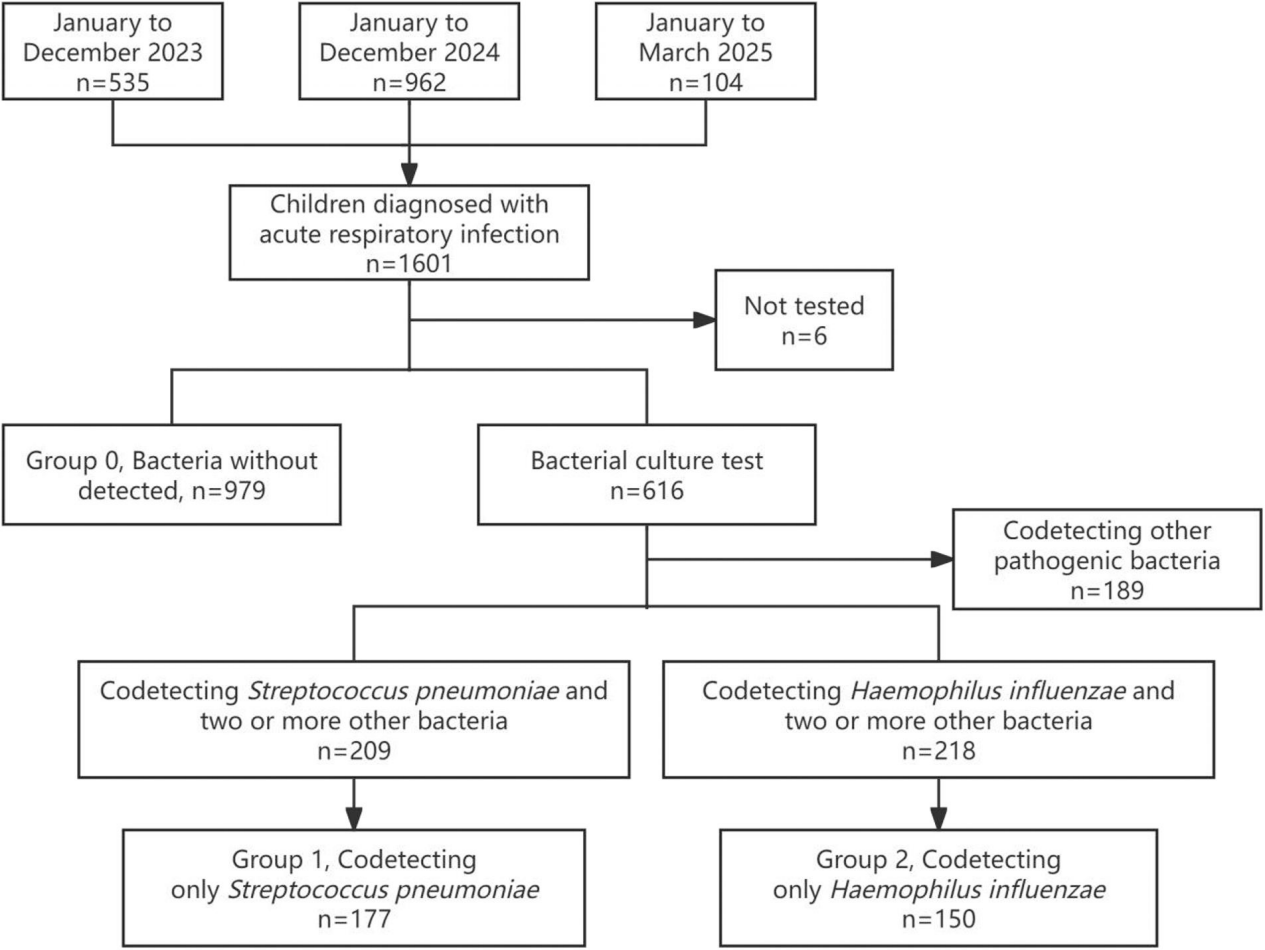
Recruitment flowchart of hospitalized children with acute respiratory infections due to RSV infection from January 2023 to march 2025.

This study included a total of 1,601 hospitalized children with acute respiratory infections: 535 in 2023, 962 in 2024, and 104 from January to March 2025. Of these, 1,596 children underwent respiratory bacterial testing; 979 showed non-bacterial growth, while 616 tested positive for bacterial codetection. Among children with codetection, 209 had *S. pneumoniae* plus at least one other bacterial species, and 177 were positive for *S. pneumoniae* alone. Additionally, 218 children had *H. influenzae* plus at least one other bacterial species, with 150 testing positive for *H. influenzae* alone. A further 189 children were codetected with other pathogens, including *Moraxella catarrhalis* (*n* = 74), *Staphylococcus aureus* (*n* = 72), *Escherichia coli* (*n* = 26), *Klebsiella pneumoniae* (*n* = 7), and others. Among these, 1,275 provided nasopharyngeal swab samples, and 31 children (who required mechanical ventilation) provided endotracheal aspirate samples, as detailed in Figure 1.

The primary focus of this study was children hospitalized with acute respiratory infections caused by RSV and codetected with *S. pneumoniae* or *H. influenzae*. To eliminate potential confounding effects from codetections with other pathogens, these cases were divided into three groups:

1. Group 0: children with RSV infection and non-bacterial detection;
2. Group 1: children with RSV infection and codetection solely with S. pneumoniae;
3. Group 2: children with RSV infection and codetection solely with H. influenzae.

### 3.1 Pairwise comparison of demographic characteristics Among the three groups

No significant differences were observed in gender distribution between the group with non-bacterial detection and the *S. pneumoniae* and *H. influenzae* codetection groups (*P* for male: 0.739 and 0.765). Age differences were not significant (*P* = 0.605, *P* = 0.243), nor was there a significant difference in the proportion of children with underlying conditions (*P* = 0.094, *P* = 0.922). The history of allergies also did not differ significantly (*P* = 0.609, *P* = 0.846), nor was there a significant difference in the proportion of exclusively breastfed children (*P* = 0.130, *P* = 0.127). However, compared to the group without bacterial detection, children in the *S. pneumoniae* and *H. influenzae* codetection groups were more likely to have a history of wheezing (*P* = 0.009, *P* = 0.045), as shown in Table 1.

**Table 1:** Pairwise comparison of demographic characteristics among three groups.

Variables	No./Total no. (%)			Group 0 vs. Group 1		Group 0 vs. Group 2	
	Group 0 (*n* = 979)	Group 1 (*n* = 177)	Group 2 (*n* = 150)	Statistic	*P*	Statistic	*P*
Male (%)^a^	601 (61.39)	111 (62.71)	94 (62.67)	*χ*² = 0.11	0.739	χ² = 0.09	0.765
Age (months, Q₁, Q₃)^b^	12.00 (5.30, 30.00)	12.00 (5.80, 21.00)	9.78 (5.29, 20.00)	*Z* = −0.52	0.605	*Z* = −1.17	0.243
Former medical history, *n* (%)^a^	74 (7.56)	20 (11.30)	11 (7.33)	χ² = 2.81	0.094	χ² = 0.01	0.922
Allergy history, *n* (%)^a^	93 (9.50)	19 (10.73)	15 (10.00)	χ² = 0.26	0.609	χ² = 0.04	0.846
Wheezing history, *n* (%)^a^	66 (6.74)	22 (12.43)	17 (11.33)	χ² = 6.90	**0**.**009**	χ² = 4.03	**0**.**045**
Breastfeed, *n* (%)^a^	612 (62.51)	100 (56.50)	84 (56.00)	χ² = 2.29	0.130	χ² = 2.33	0.127

Bold values denote statistical significance (*p* < 0.05).

a *P* < 0.05 The categorical variables were compared using the chi-square test and summarized as percentages.

b *P* < 0.05 The Mann–Whitney *U*-test (*Z*-test statistic) was used to compare non-normally distributed continuous variables, reported as quartiles.

### 3.2 Pairwise comparison of clinical characteristics and laboratory test results Among the three groups

#### 3.2.1 Clinical characteristics

There were no significant differences found in the incidence of cough (*P* = 1), wheezing (*P* = 0.107), or difficulty breathing (*P* = 0.504) between the group with non-bacterial detection and the *S. pneumoniae* codetection group. However, the proportion of children with fever was significantly higher in the *S. pneumoniae* codetection group (*P* = 0.040). Similarly, compared to the group with non-bacterial detection, the H. influenzae codetection group had no significant differences in fever (*P* = 0.098) or cough (*P* = 1), but significantly higher rates of wheezing (*P* = 0.046) and difficulty breathing (*P* < 0.001). Furthermore, children codetected with *S. pneumoniae* or *H. influenzae* had a significantly higher proportion of extrapulmonary manifestations (*P* = 0.039, *P* = 0.013).

#### 3.2.2 Laboratory test results

Comparison Between the non-bacterial detection group and the *S. pneumoniae* codetection group:

There were no significant differences in the following variables between the non-bacterial detection group and the *S. pneumoniae* codetection group: hemoglobin (*P* = 0.146), platelets (*P* = 0.698), ferritin (*P* = 0.554), alanine aminotransferase (*P* = 0.093), aspartate aminotransferase (*P* = 0.448), creatine kinase (*P* = 0.937), creatine kinase MB (*P* = 0.775), lactate dehydrogenase (*P* = 0.330), blood urea nitrogen (*P* = 0.684), creatinine (*P* = 0.810), uric acid (*P* = 0.057), sodium (*P* = 0.669), potassium (*P* = 0.451), calcium (*P* = 0.499), prothrombin time (*P* = 0.803), activated partial thromboplastin time (*P* = 0.294), D-dimer (*P* = 0.408), and antithrombin III (*P* = 0.562). However, the *S. pneumoniae* codetection group showed significantly higher levels of white blood cells (WBC) (*P* = 0.005), neutrophil-to-lymphocyte ratio (NLR) (*P* = 0.021), C-reactive protein (CRP) (*P* = 0.011), procalcitonin (PCT) (*P* = 0.018), and interleukin-6 (IL-6) (*P* < 0.001) compared to the non-bacterial detection group.

Comparison Between the non-bacterial detection group and the *H. influenzae* codetection group:

There were no significant differences in the following variables between the non-bacterial detection group and the *H. influenzae* codetection group: hemoglobin (*P* = 0.076), ferritin (*P* = 0.053), alanine aminotransferase (*P* = 0.307), aspartate aminotransferase (*P* = 0.451), creatine kinase (*P* = 0.230), creatine kinase MB (*P* = 0.260), lactate dehydrogenase (*P* = 0.483), blood urea nitrogen (*P* = 0.504), creatinine (*P* = 0.172), uric acid (*P* = 0.366), sodium (*P* = 0.989), potassium (*P* = 0.345), calcium (*P* = 0.693), prothrombin time (*P* = 0.648), activated partial thromboplastin time (*P* = 0.340), and antithrombin III (*P* = 0.613). However, the *H. influenzae* codetection group had significantly higher levels of WBC (*P* < 0.001), NLR (*P* < 0.001), CRP (*P* < 0.001), PCT (*P* < 0.001), IL-6 (*P* < 0.001), platelet count (PLT) (*P* = 0.003), and D-dimer (*P* < 0.001) compared to the non-bacterial detection group, as shown in Table 2.

**Table 2:** Pairwise comparison of clinical features and laboratory test results among three groups.

Variables	No./Total no. (%)			Group 0 vs. Group 1		Group 0 vs. Group 2	
	Group 0 (*n* = 979)	Group 1 (*n* = 177)	Group 2 (*n* = 150)	Statistic	*P*	Statistic	*P*
Fever, *n* (%)^a^	624 (63.74)	127 (71.75)	106 (70.67)	χ² = 4.23	**0**.**040**	χ² = 2.73	0.098
Cough, *n* (%)^a^	978 (99.90)	177 (100.00)	150 (100.00)	–	1.000	–	1.000
Wheezing, *n* (%)^a^	227 (23.19)	51 (28.81)	46 (30.67)	χ² = 2.60	0.107	χ² = 3.97	**0**.**046**
Dyspnea, *n* (%)^a^	49 (5.01)	11 (6.21)	19 (12.67)	χ² = 0.45	0.504	χ² = 13.49	**<**.**001**
Non-respiratory Diagnoses, *n* (%)^a^	167 (17.06)	44 (24.86)	36 (24.00)	χ² = 4.25	**0**.**039**	χ² = 6.11	**0**.**013**
WBC (×10^9^/L), M (Q₁, Q₃)^b^	8.55 (6.54, 10.92)	9.46 (7.30, 11.81)	10.60 (8.46, 13.05)	*Z* = −2.81	**0**.**005**	*Z* = −6.22	**<**.**001**
NLR, M (Q₁, Q₃)^b^	0.64 (0.34, 1.16)	0.74 (0.46, 1.17)	0.92 (0.54, 1.79)	*Z* = −2.30	**0**.**021**	*Z* = −4.59	**<**.**001**
HGB (g/L), M (Q₁, Q₃)^b^	114.00 (106.00, 121.00)	112.00 (105.00, 120.00)	112.50 (103.25, 119.00)	*Z* = −1.45	0.146	*Z* = −1.77	0.076
CRP (mg/L), M (Q₁, Q₃)^b^	1.40 (0.39, 5.70)	2.57 (0.51, 7.36)	6.86 (0.69, 16.53)	*Z* = −2.54	**0**.**011**	*Z* = −6.70	**<**.**001**
PCT (ng/mL), M (Q₁, Q₃)^b^	0.10 (0.05, 0.15)	0.11 (0.06, 0.19)	0.12 (0.07, 0.33)	*Z* = −2.37	**0**.**018**	*Z* = −4.55	**<**.**001**
SF(ng/mL), M (Q₁, Q₃)^b^	60.32 (28.09, 105.60)	64.07 (33.25, 102.30)	75.64 (30.26, 123.34)	*Z* = −0.59	0.554	*Z* = −1.93	0.053
IL-6 (pg/mL), M (Q₁, Q₃)^b^	7.00 (2.50, 15.90)	15.03 (5.80, 31.57)	15.86 (5.06, 37.10)	*Z* = −6.75	**<**.**001**	*Z* = −5.81	**<**.**001**
PLT (×10^9^/L), M (Q₁, Q₃)^b^	364.00 (289.00, 435.50)	364.00 (283.00, 424.00)	405.00 (318.25, 460.50)	*Z* = −0.39	0.698	*Z* = −2.92	**0**.**003**
ALT (U/L), (X ± s)^c^	28.98 ± 28.77	25.18 ± 20.31	26.36 ± 32.52	*t* = 1.683	0.093	*t* = 1.021	0.307
AST (U/L), (X ± s)^c^	51.06 ± 120.50	44.18 ± 14.88	43.63 ± 18.14	*t* = 0.758	0.448	*t* = 0.754	0.451
CK (U/L), (X ± s)^c^	116.39 ± 100.15	115.72 ± 111.40	106.11 ± 77.69	*t* = 0.080	0.937	*t* = 1.202	0.230
CK-MB (U/L), (X ± s)^c^	31.04 ± 57.51	29.79 ± 17.70	25.72 ± 12.94	*t* = 0.286	0.775	*t* = 1.128	0.260
LDH (U/L), (X ± s)^c^	304.81 ± 139.86	315.29 ± 72.00	312.99 ± 71.51	*t* = −.974	0.330	*t* = −.702	0.483
BUN (mmol/L), (X ± s)^c^	3.68 ± 11.65	3.32 ± 4.44	4.46 ± 21.45	*t* = 0.406	0.684	*t* = −.669	0.504
Cr(umol/L),M (Q₁, Q₃)^b^	22.80 (18.40, 28.00)	22.60 (18.70, 27.80)	22.20 (17.40, 26.80)	*Z* = −0.24	0.810	*Z* = −1.37	0.172
UA (umol/L), M (Q₁, Q₃)^b^	228.65 (188.93, 283.05)	245.70 (198.00, 299.50)	237.65 (193.05, 288.65)	*Z* = −2.38	0.057	*Z* = −0.90	0.366
Na (mmol/L), (X ± s)^c^	137.89 ± 14.16	138.35 ± 1.80	137.87 ± 2.40	*t* = −.427	0.669	*t* = 0.014	0.989
K (mmol/L), (X ± s)^c^	4.51 ± 0.62	4.46 ± 0.54	4.75 ± 0.57	*t* = 0.753	0.451	*t* = 0.687	0.345
Ca (mmol/L), (X ± s)^c^	2.32 ± 0.17	2.31 ± 0.13	2.33 ± 0.16	*t* = 0.677	0.499	*t* = 0.394	0.693
PT (s), (X ± s)^c^	11.8 ± 4.20	11.88 ± 1.52	11.64 ± 1.24	*t* = −.249	0.803	*t* = 0.457	0.648
APTT (s),M (Q₁, Q₃)^b^	30.20 (27.10, 33.22)	29.80 (26.80, 32.70)	29.95 (27.42, 34.95)	*Z* = −1.05	0.294	*Z* = −0.95	0.340
D-Dimer (ug/mL),M (Q₁, Q₃)^b^	0.32 (0.22, 0.44)	0.33 (0.23, 0.43)	0.39 (0.27, 0.51)	*Z* = −0.83	0.408	*Z* = −4.16	**<**.**001**
AT-III (%), M (Q₁, Q₃)^c^	104.90 (96.15, 111.20)	105.60 (97.10, 112.50)	104.95 (97.90, 111.70)	*Z* = −0.58	0.562	*Z* = −0.51	0.613

WBC, White blood cell count; NLR, Neutrophil-to-lymphocyte ratio; HGB, Hemoglobin; CRP, C-reactive protein; PCT, Procalcitonin; SF, Serum ferritin; IL-6, Interleukin 6; PLT, Platelet; ALT, Alanine aminotransferase; AST, Aspartate aminotransferase; CK, Creatine kinase; CK-MB, Creatine kinase isoenzymes; LDH, Lactate dehydrogenase; BUN, Blood urea nitrogen; Cr, Creatinine; UA, Uric acid; Na, Serum Sodium; K, Serum K; Ca, Serum calcium; PT, Prothrombin time; APTT, Activated partial thromboplastin time; AT-III, Antithrombin III.

Bold values denote statistical significance (*p* < 0.05).

a *P* < 0.05 The categorical variables were compared using the chi-square test and summarized as percentages.

b *P* < 0.05 The Mann–Whitney *U*-test (*Z*-test statistic) was used to compare non-normally distributed continuous variables, reported as quartiles.

c *P* < 0.05 The normally distributed data were analyzed using independent *t*-tests with results presented as mean ± standard deviation (SD).

#### 3.2.3 Pairwise comparison of treatment and outcomes Among the three groups

Compared to the group without bacterial detection, children in the *S. pneumoniae* and *H. influenzae* codetection groups exhibited a significantly higher proportion of invasive/non-invasive respiratory support (*P* = 0.014, *P* < 0.001), higher PICU admission rates (*P* = 0.032, *P* = 0.003), and longer hospital stays (*P* = 0.022, *P* = 0.033). However, there was no significant difference in mortality rates among the three groups (*P* = 1), as shown in Table 3.

**Table 3:** Pairwise comparison of treatment and outcomes among three groups.

Variables		No./Total no. (%)			Group 0 vs. Group 1		Group 0 vs. Group 2	
		Group 0 (*n* = 979)	Group 1 (*n* = 177)	Group 2 (*n* = 150)	Statistic	*P*	Statistic	*P*
Respiratory support, *n* (%), M (Q₁, Q₃)^b^	Invasive, *n* (%)	15 (1.53)	9 (5.08)	7 (4.67)	–	**0.014**	–	**<**.**001**
	Non-invasive, *n* (%)	6 (0.61)	1 (0.56)	5 (3.33)				
Admitted to the PICU, *n* (%)^a^		92 (9.40)	26 (14.69)	26 (17.33)	χ² = 4.58	**0.032**	χ² = 8.75	**0.003**
Hospitalization duration (d), M (Q₁, Q₃)^b^		7.00 (6.00, 8.00)	7.00 (6.00, 8.00)	7.00 (6.00, 8.00)	*Z* = −2.29	**0.022**	*Z* = −2.13	**0.033**
Mortality, *n* (%)^a^		0 (0.00)	0 (0.00)	0 (0.00)	–	1	–	1

PICU, Pediatric intensive care unit.

Bold values denote statistical significance (*p* < 0.05).

a *P* < 0.05 The categorical variables were compared using the chi-square test and summarized as percentages.

b *P* < 0.05 The Mann–Whitney *U*-test (*Z*-test statistic) was used to compare non-normally distributed continuous variables, reported as quartiles.

#### 3.2.4 Early predictive indicators of codetection with *S. pneumoniae* and *H. influenzae* in Children with RSV

The logistic regression analysis was performed to compare children with RSV/*S. pneumoniae* codetection and those without bacterial detection. Univariate analysis revealed that a history of wheezing, fever, extrapulmonary manifestations, invasive respiratory support, length of hospitalization, WBC count, and IL-6 were significant risk factors for the presence of *S.pneumoniae* in the respiratory tract. After including statistically significant factors from the univariate analysis into the multivariate regression model, the results showed that a history of wheezing (OR = 1.87, 95% CI 1.11–3.16, *P* = 0.019) and IL-6 levels (OR = 1.01, 95% CI 1.01–1.01, *P* < 0.001) were independent risk factors for *S. pneumoniae* codetection, serving as early predictive indicators, as shown in Table 4.

**Table 4:** Risk factor analysis of codetection *S. pneumoniae* in children with RSV infection.

Variables	Univariate analysis					Multivariate analysis				
	*β*	S.E	*Z*	*P*	OR (95%CI)	*β*	S.E	*Z*	*P*	OR (95%CI)
Wheezing History (yes vs. no)	0.67	0.26	2.58	**0**.**010**	1.96 (1.18–3.28)	0.63	0.27	2.34	**0**.**019**	1.87 (1.11–3.16)
Fever (yes vs. no)	0.37	0.18	2.05	**0**.**041**	1.45 (1.02–2.06)	0.30	0.18	1.61	0.107	1.35 (0.94–1.93)
Non-respiratory Diagnoses (yes vs. no)	0.48	0.19	2.46	**0**.**014**	1.61 (1.10–2.35)	0.12	0.23	0.55	0.584	1.13 (0.73–1.77)
Respiratory support										
Invasive	1.24	0.43	2.88	**0**.**004**	3.44 (1.48–7.99)	0.46	0.59	0.77	0.442	1.58 (0.49–5.04)
Non-invasive	−0.04	1.08	−0.04	0.967	0.96 (0.11–7.99)	−0.58	1.13	−0.51	0.607	0.56 (0.06–5.14)
Hospitalization duration (d)	0.09	0.03	3.37	**<**.**001**	1.09 (1.04–1.15)	0.06	0.04	1.55	0.121	1.06 (0.98–1.14)
WBC (×10^9^/L)	0.03	0.02	2.10	**0**.**036**	1.03 (1.01–1.07)	0.02	0.02	0.92	0.357	1.02 (0.98–1.05)
IL-6 (pg/mL)	0.01	0.00	4.58	**<**.**001**	1.01 (1.01–1.01)	0.01	0.00	4.19	**<**.**001**	1.01 (1.01–1.01)

WBC, White blood cell count; IL-6, Interleukin 6.

Bold values denote statistical significance (*p* < 0.05).

Similarly, logistic regression analysis was conducted for children codetected with *H. influenzae* and compared to those without bacterial detection. Univariate analysis indicated that a history of wheezing, wheezing, respiratory distress, extrapulmonary manifestations, respiratory support (both invasive and non-invasive), age, length of hospitalization, WBC count, NLR, CRP, PLT, and IL-6 were significant risk factors for the presence of *H. influenzae* codetection. When these statistically significant factors from the univariate analysis were included in the multivariate regression model, the results showed that younger age (OR = 0.98, 95% CI 0.97–0.99, *P* = 0.008), higher WBC (OR = 1.07, 95% CI 1.02–1.11, *P* = 0.002), higher CRP (OR = 1.03, 95% CI 1.02–1.04, *P* < 0.001), and elevated IL-6 levels (OR = 1.01, 95% CI 1.01–1.01, *P* = 0.017) were independent risk factors for *H. influenzae* codetection, serving as early predictive indicators, as shown in Table 5.

**Table 5:** Risk factor analysis of codetection *H. influenzae* in children with RSV infection.

Variables	Univariate analysis					Multivariate analysis				
	*β*	S.E	*Z*	*P*	OR (95%CI)	*β*	S.E	*Z*	*P*	OR (95%CI)
Wheezing History (yes vs. no)	0.57	0.29	1.98	**0**.**047**	1.77 (1.01–3.11)	0.48	0.32	1.51	0.131	1.62 (0.87–3.01)
Wheezing (yes vs. no)	0.38	0.19	1.98	**0**.**047**	1.47 (1.01–2.14)	0.02	0.25	0.08	0.933	1.02 (0.63–1.65)
Dyspnea (yes vs. no)	1.01	0.29	3.54	**<**.**001**	2.75 (1.57–4.82)	−0.06	0.50	−0.12	0.907	0.94 (0.35–2.53)
Non-respiratory Diagnoses (yes vs. no)	0.43	0.21	2.05	**0**.**040**	1.54 (1.02–2.31)	0.01	0.26	0.04	0.969	1.01 (0.61–1.68)
Respiratory support										
Invasive	1.18	0.47	2.52	**0**.**012**	3.24 (1.30–8.09)	0.87	0.72	1.22	0.224	2.39 (0.59–9.70)
Non-invasive	1.76	0.61	2.87	**0**.**004**	5.79 (1.74–19.21)	1.19	0.83	1.43	0.153	3.29 (0.64–16.88)
Age (months)	−0.01	0.00	−2.28	**0**.**023**	0.99 (0.98–0.99)	−0.02	0.01	−2.64	**0**.**008**	0.98 (0.97–0.99)
Hospitalization duration (d)	0.08	0.03	2.89	**0**.**004**	1.09 (1.03–1.15)	−0.03	0.05	−0.53	0.593	0.97 (0.89–1.07)
WBC (×10^9^/L)	0.10	0.02	5.43	**<**.**001**	1.11 (1.07–1.15)	0.06	0.02	3.09	**0**.**002**	1.07 (1.02–1.11)
NLR	0.11	0.04	2.55	**0**.**011**	1.11 (1.02–1.21)	0.07	0.04	1.51	0.130	1.07 (0.98–1.16)
CRP (mg/L)	0.03	0.01	6.20	**<**.**001**	1.04 (1.02–1.05)	0.03	0.01	4.36	**<**.**001**	1.03 (1.02–1.04)
IL-6(pg/mL)	0.01	0.00	4.61	**<**.**001**	1.01 (1.01–1.01)	0.01	0.00	2.39	**0**.**017**	1.01 (1.01–1.01)
PLT (ng/mL)	0.01	0.00	3.15	**0**.**002**	1.01 (1.01–1.01)	0.00	0.00	1.22	0.223	1.00 (1.00–1.00)

WBC, White blood cell count; NLR, Neutrophil-to-lymphocyte ratio; CRP, C-reactive protein; IL-6, Interleukin 6; PLT, Platelet.

Bold values denote statistical significance (*p* < 0.05).

## 4 Discussion

This retrospective study included 1,601 hospitalized children with RSV infection and compared the demographic characteristics, clinical presentations, laboratory findings, and outcomes between children with codetection of *S. pneumoniae* and those with codetection of *H. influenzae*. Additionally, the study explored risk factors associated with codetection of each bacterium, aiming to provide early clinical predictive indicators before pathogen detection results become available.

In this study, the codetection rate of bacteria in children with RSV infection was found to be 38.48%. In a hospital based-study in Finland, bacterial infections were observed in 39% of RSV-positive children (26).However, a well-established 20 sentinel healthcare facilities surveillance study in Portugal showed that only 20.4% of children hospitalized with RSV had bacterial infections (27). A study by Chinese scholars in Wenzhou, involving 1,063 hospitalized RSV-infected children, reported that only 20.7% of RSV-positive children had bacterial co-infections (28). Research from Vietnam shows that the proportion of RSV-infected children with bacterial co-infections is 15.7% (29). A study in Taipei indicated that, among 620 hospitalized children with RSV pneumonia, 38.6% of those under the age of 1 had bacterial infections (30), which is consistent with our findings. Consistent with existing literature, the highest rate of bacterial detection was for *S. pneumoniae*, followed by *H. influenzae* (28). We observed that children with a history of wheezing had a higher likelihood of having S. pneumoniae and H. influenzae detected in their respiratory secretions when infected with RSV. Additionally, when both of these bacteria were codetected, the children's levels of WBC, NLR, CRP, PCT, and IL-6 were significantly higher compared to those without bacterial detection. Moreover, children with codetection of *H. influenzae* were more likely to experience wheezing and respiratory distress than those in the group without bacterial detection. Studies have shown that RSV infection promotes the development of asthma through both direct and indirect mechanisms. The direct effect involves damage to airway epithelial cells, as RSV primarily targets alveolar and ciliated epithelial cells, leading to cell death through necrosis or apoptosis (31). The indirect effect is related to changes in the immune response, with RSV skewing the immune response towards a pathogenic Th2 polarization, which increases asthma susceptibility (32). Recent studies have revealed that during RSV infection, the respiratory microbiota undergoes structural and diversity changes that favor the growth of species such as *Moraxella*, *Haemophilus*, *Staphylococcus aureus*, and *Streptococcus* species. In a prospective observational cohort study, 136 children with RSV-induced bronchiolitis were included and compared to age-matched healthy controls, those with RSV bronchiolitis had higher rates of *H. influenzae*, *Moraxella catarrhalis*, *S. pneumoniae*, and *Staphylococcus aureus* colonization in their nasopharynx. Additionally, a higher percentage of neutrophils in the blood and increased plasma levels of IL-8 and IL-6 were associated with the presence of Gram-negative bacteria (33). This could be related to an enhanced mucosal pro-inflammatory response from the presence of *S. pneumoniae* or *H. influenzae* in the nasopharynx of children (34–36). Mechanistically, RSV infection activates T cell responses to inhaled allergens and increases the activity of Th2 cytokines (such as IL-4, IL-5, and IL-13) in the airway mucosa (31), a process that is associated with the overexpression of genes linked to Toll-like receptor (TLR) signaling and neutrophil recruitment and activation. When a nasopharyngeal microbiota rich in *H.influenzae* and *Streptococcus* encounters an RSV infection, it may lead to more severe clinical manifestations and exacerbate the host's inflammatory immune response (37), resulting in heightened cell reactivity and mucus metaplasia (31). Furthermore, NLR, which reflects systemic inflammatory immune responses, offers better sensitivity and specificity than WBC (38).

It is noteworthy that children codetected with *H. influenzae* exhibited significant increases in platelet count (PLT) and D-dimer levels. This may be related to the activation of the coagulation cascade, as D-dimer (a degradation product of cross-linked fibrin) often rises during viral infections. Pathological elevation of D-dimer may reflect the activation of inflammatory responses and the coagulation cascade, suggesting that D-dimer could serve as an indicator for assessing the severity of infection (39). While the proportion of children with codetection of *S. pneumoniae* is higher, the presence of *H. influenzae* codetection in these patients may be associated with more pronounced abnormalities in various biomarkers.

The codetection of *S. pneumoniae* and *H. influenzae* significantly increased the need for respiratory support, PICU admission rates, and hospital length of stay. A study involving children admitted to the PICU due to RSV-induced bronchiolitis, which included 165 children who received mechanical ventilation, found that children with bacterial codetections required longer ventilation support and hospitalization. This result aligns with our study's findings (40). However, our study also found that, despite *S. pneumoniae* being more commonly detected than *H. influenzae*, the latter was associated with more severe clinical manifestations, such as wheezing and respiratory distress, as well as higher levels of laboratory abnormalities, including elevated platelet count (PLT) and D-dimer. Nevertheless, since no fatalities were observed in either group, our study did not detect significant differences in final outcomes. Further research is needed to investigate whether the more pronounced abnormal biomarkers associated with *H. influenzae* lead to worse clinical outcomes.

Based on the above findings, we conducted a logistic regression analysis to assess the clinical features and laboratory results of RSV-infected children with co-existing *S. pneumoniae* and *H. influenzae*. The multivariate analyses revealed that a history of wheezing and significantly elevated IL-6 levels were predictive of the presence of *S. pneumoniae* in the respiratory tract. Additionally, younger age, along with higher levels of WBC, CRP, and IL-6, was associated with a higher likelihood of codetection with *H. influenzae*. In summary, the presence of either *S. pneumoniae* or *H. influenzae* substantially increased the likelihood of PICU admission and the need for mechanical ventilation support, while also potentially prolonging the hospital stay.

Current studies indicate that there are no specific clinical symptoms or diagnostic tests that can reliably identify patients with positive bacterial cultures. Therefore, for children with RSV-induced bronchiolitis, especially those requiring admission to the PICU, early microbial testing of tracheal aspirates is recommended. However, since test results take time and may be affected by prior antibiotic use or limitations in diagnostic methods, false negatives may occur. Consequently, there is an urgent need for predictive indicators that can help identify codetection bacterial infections, thereby increasing clinical awareness and aiding timely and appropriate treatment. This could even guide the selection of antibiotics for anti-infective therapy. Furthermore, for children with a low likelihood of codetection, delaying the use of antibiotics could be considered, as overuse of antibiotics can exacerbate disease severity, such as prolonging oxygen therapy or increasing the risk of pulmonary consolidation (33). Therefore, reliable clinical predictive markers are crucial not only to prevent delays in treatment but also to reduce the unnecessary overuse of antibiotics.

## 5 Limitations

This study has several limitations: 1. It is a retrospective single-center study, which limits the generalizability of the findings. To mitigate this issue, we made efforts to maximize the sample size and addressed missing data using median and mode imputation methods. 2. Due to the absence of RSV infection testing technology before January 2023, and the time required for data collection, analysis, and reporting, the data collected in this study ended in March 2025, meaning recent data were not included. 3. Due to limitations in the testing methods, the current study only included the cytokine IL-6, and testing for other cytokines has not been fully implemented. 4. Our study detected bacteria from nasopharyngeal swabs, which cannot distinguish between true lower respiratory tract co-infection and upper respiratory tract colonization. Future studies using lower respiratory tract samples (e.g., bronchoalveolar lavage) or rigorous clinical criteria (e.g., procalcitonin > 0.5 ng/mL, radiographic evidence of pneumonia) are needed to validate whether these codetections represent clinically meaningful co-infections.

## 6 Conclusions

Compared to children with RSV infection alone, those with codetections of *S. pneumoniae* or *H. influenzae* exhibit significantly elevated inflammatory markers, especially IL-6. These children are more likely to require PICU admission, respiratory support, and experience longer hospital stays.

## Data Availability

The raw data supporting the conclusions of this article will be made available by the authors, without undue reservation.
